# Supplementary material for: Parasites trigger epithelial cell crosstalk to drive gut–brain signalling
Source: Nature. 2026 Mar 25;653(8114):465–73. doi: 10.1038/s41586-026-10281-5 (PMC13171452; doi:10.1038/s41586-026-10281-5)
Supplement: Supplementary file 1 — Reporting Summary [file 41586_2026_10281_MOESM1_ESM.pdf]

nature portfolio

Corresponding author(s): Kouki K. Touhara and David Julius

Last updated by author(s): 1/25/2026

Reporting Summary

Nature Portfolio wishes to improve the reproducibility of the work that we publish. This form provides structure for consistency and transparency in reporting. For further information on Nature Portfolio policies, see our Editorial Policies and the Editorial Policy Checklist.

Statistics

For all statistical analyses, confirm that the following items are present in the figure legend, table legend, main text, or Methods section.

n/a	Confirmed	
☐	☒	The exact sample size (*n*) for each experimental group/condition, given as a discrete number and unit of measurement
☐	☒	A statement on whether measurements were taken from distinct samples or whether the same sample was measured repeatedly
☐	☒	The statistical test(s) used AND whether they are one- or two-sided<br>*Only common tests should be described solely by name; describe more complex techniques in the Methods section.*
☐	☒	A description of all covariates tested
☐	☒	A description of any assumptions or corrections, such as tests of normality and adjustment for multiple comparisons
☐	☒	A full description of the statistical parameters including central tendency (e.g. means) or other basic estimates (e.g. regression coefficient) AND variation (e.g. standard deviation) or associated estimates of uncertainty (e.g. confidence intervals)
☐	☒	For null hypothesis testing, the test statistic (e.g. *F*, *t*, *r*) with confidence intervals, effect sizes, degrees of freedom and *P* value noted<br>*Give P values as exact values whenever suitable.*
☒	☐	For Bayesian analysis, information on the choice of priors and Markov chain Monte Carlo settings
☒	☐	For hierarchical and complex designs, identification of the appropriate level for tests and full reporting of outcomes
☒	☐	Estimates of effect sizes (e.g. Cohen's *d*, Pearson's *r*), indicating how they were calculated

Our web collection on statistics for biologists contains articles on many of the points above.

Software and code

Policy information about availability of computer code

Data collection	Immunofluorescence images of tissue sections and cells were captured on a Nikon CSU-W1 spinning disk confocal microscope run by the Micro-Manager software (v2.0). Serotonin and acetylcholine sensor images were acquired through a Leica SP8 confocal microscope run by the LAS X software (v3.5.5.19976) or an upright microscope equipped with a Grasshopper 3 (FLIR) camera run by the Micro-Manager software (v2.0). Patch-clamp recordings were made by using a Digidata 1550B digitizer (Molecular Devices) connected to the pClamp software (v10.7). Nerve fiber recordings were made by using a 1401 interface (CED, Cambridge, UK) run by Spike2 software (V.5.18.). Optogenetic activation of nerve fibers was delivered using a High Power Fiber-Coupled LED Light Source (model BLS-FCS-0470-10) and Multimode Fiber Patchcords (Numerical aperture: 0.39 NA, Core size: 400 µm. Catalog # FPC-0400-39-025MA-BP, Mightex, Pleasanton, CA 94566, US). Single cell RT-PCR was performed on an Applied Biosystems® 7500 Real-Time PCR System. Calcium imaging of dissociated nodose neurons were made by using a Olympus IX71 microscope equipped with a CCD camera (Retiga ELECTRO) in conjunction with a Sutter Lambda 10-3 wavelength switcher and the Chroma filter set no. 49011 (ET480/40x (Ex), T510lpxrxt (BS), ET535/50m (Em)). Behaviour was measured using a Behavioural spectrometer (Behavior Sequencer, Behavioral Instruments, NJ and BiObserve, DE). Abdominal electronic Von Frey Hair test was performed in the clear plexiglas observation chamber (BSBIOPVF, Panlab, Spain).

Data analysis

We used the Fiji software (NIH, v2.14.) to generate maximal intensity projections and generate GCaMP and serotonin/acetylcholine sensor deltaF/F images and traces. The Clampfit software (v11.2.2.17, Molecular Devices) was used to analyze patch-clamp recording data. Immunofluorescence images were analyzed with Leica LAS Lite 4.0 (Leica Microsystems) or Fiji software v2.14 (NIH). Statistical analyses were done by using the Prism software (GraphPad, v8.4.3). Nerve fiber recordings were analyzed using Spike2 software (V.5.18). Single cell RT-PCR curves were analysed using 7500 Software v.2.06 from Life Technologies. Calcium imaging of dissociated neurons were analyzed using Metafluor software (Molecular Devices, V.7.8.0.0). Behaviour measured using a Behavioural spectrometer was analyzed by a computerized video tracking software (Viewer3, BiObserve, DE). Von Frey Hair data was analyzed with BIOCIS Force Ramp Software. Published single-cell RNA sequencing datasets were analyzed with Seurat 5.0.1. on R 4.4.1.

For manuscripts utilizing custom algorithms or software that are central to the research but not yet described in published literature, software must be made available to editors and reviewers. We strongly encourage code deposition in a community repository (e.g. GitHub). See the Nature Portfolio guidelines for submitting code & software for further information.

Data

Policy information about availability of data

All manuscripts must include a data availability statement. This statement should provide the following information, where applicable:

- Accession codes, unique identifiers, or web links for publicly available datasets
- A description of any restrictions on data availability
- For clinical datasets or third party data, please ensure that the statement adheres to our policy

All data generated or analyzed during this study are included in the manuscript.

Research involving human participants, their data, or biological material

Policy information about studies with human participants or human data. See also policy information about sex, gender (identity/presentation), and sexual orientation and race, ethnicity and racism.

Reporting on sex and gender	N/A
Reporting on race, ethnicity, or other socially relevant groupings	N/A
Population characteristics	N/A
Recruitment	N/A
Ethics oversight	N/A

Note that full information on the approval of the study protocol must also be provided in the manuscript.

Field-specific reporting

Please select the one below that is the best fit for your research. If you are not sure, read the appropriate sections before making your selection.

☒ Life sciences ☐ Behavioural & social sciences ☐ Ecological, evolutionary & environmental sciences

For a reference copy of the document with all sections, see nature.com/documents/nr-reporting-summary-flat.pdf

Life sciences study design

All studies must disclose on these points even when the disclosure is negative.

Sample size

For statistical comparisons, sample size was selected based on power calculations performed with reference to previous or present experiments carried out in our laboratory and in the field. For patch-clamp recording experiments, we collected data from 6 cells. For Ca2+ imaging experiments, we collected data from 6-15 cells. For biosensor experiments, we collected data from 6-21 organoids. For ex vivo serotonin sensor imaging comparing crypts vs. villi, we collected data from 9-17 samples in 2 mice. For other ex vivo serotonin sensor imaging, we collected images from 136-710 crypts from 3-5 animals. For cFOS staining, we collected data from 4-11 brainstem slices from 4-11 animals. For food intake measurements with IL-25 injected mice, we collected data from 10-15 mice (a mixture of males and females). For food intake measurements from helminth-infected mice, we collected data from 10 mice (a mixture of males and females). For nerve fiber recordings, we collected data from 990 nerve fibers recorded from 41 mice (a mixture of males and females). For RT-PCR, we collected data from 43 cells isolated from 3 mice. For calcium imaging we collected data from 68 cells from 6 male mice. We performed behavioral measurements for two studies. 1) Oral gavage of vehicle/Agonist 39 from 15 C57/BL6 mice. 2) I.P administration of vehicle/IL-25 from 64 WT mice, 32PFTox+ mice, 36 Pou2f3-/- mice and 30 Vilcre;Chatflox/flox mice (a mixture of males and females). Von Frey Hair measurements were performed with 29 WT mice. Nesting behavior was measured from 30 WT mice.

Data exclusions

For Ca2+ imaging and acetylcholine/serotonin sensor experiments, cells and tissues that showed substantial movements during imaging or

Data exclusions	abnormal high K+ response were not analyzed. For patch-clamp recording, we selected recordings that were made at an access resistance <15 MΩ and showed no drifting or excessive noise, which are common criteria for whole-cell patch-clamp recordings. For single cell RT-PCR, 5 cells were excluded, 4 cells that were not positive for Tubb3 and 1 cell that was positive for GFAP expression. For calcium imaging, only cells that displaced robust responses to high KCl were included in the analysis.
Replication	For patch-clamp recording, ex vivo serotonin sensor imaging, serotonin and acetylcholine biosensor experiments, nerve fiber recordings and Ca2+ imaging experiments, we did not repeat the same stimulation on the same cell or tissue slice. Instead, we repeated the experiments on multiple cells or tissues from multiple animals and pooled the data for statistical analysis. All experiments involving multiple cohorts were routinely assessed on different days. Replication was successful on biosensor imaging, calcium imaging, patch-clamp recordings, afferent recordings, in situ hybridization, and immunohistological experiments. Behavioral tests were conducted on multiple groups of littermates or age-matched mice at different times depending on the availability of animals, and data from all groups were analyzed collectively.
Randomization	Genetically modified mice or control animals (littermates or age-matched mice) were randomly selected for histological, Ca2+ imaging, serotonin sensor imaging, and patch-clamp recording experiments.
Blinding	Experimenter was blinded for all behavioral tests. Where possible, experimenter was blinded from the genotype or drug treatment information when performing quantifications, including event counting, and intensity measurements.

Reporting for specific materials, systems and methods

We require information from authors about some types of materials, experimental systems and methods used in many studies. Here, indicate whether each material, system or method listed is relevant to your study. If you are not sure if a list item applies to your research, read the appropriate section before selecting a response.

Materials & experimental systems

n/a	Involved in the study	
☐	☒	Antibodies
☐	☒	Eukaryotic cell lines
☒	☐	Palaeontology and archaeology
☐	☒	Animals and other organisms
☒	☐	Clinical data
☒	☐	Dual use research of concern
☒	☐	Plants

Methods

n/a	Involved in the study	
☒	☐	ChIP-seq
☒	☐	Flow cytometry
☒	☐	MRI-based neuroimaging

Antibodies

Antibodies used

Target and Conjugate, Host, Dilution, Manufacturer, Catalog #, RRID

Primary antibodies:
GFP, Chicken, 1:500, Abcam, ab13970, AB_300798
DCAMKL1, Rabbit, 1:250, Abcam, ab37994, AB_873538
cFOS, Rat, 1:300, Synaptic System, 226008, AB_2891278
CCK-8, Guinea pig, 1:500, Synaptic System,, 438004, AB_2814938

Secondary antibodies:
Rabbit IgG-Alexa Fluor 647, Goat, 1:500, Thermo Fisher Scientific, A-21244, AB_2535812
Rat IgG-Alexa Fluor 647, Goat, 1:500, Thermo Fisher Scientific, A-21247, AB_141778
Rabbit IgG-Alexa Fluor 568, Goat, 1:500, Thermo Fisher Scientific, A-11036, AB_10563566
Rabbit IgG-Alexa Fluor 488, Goat, 1:500, Thermo Fisher Scientific, A-11034, AB_2576217
Chicken IgY-Alexa Fluor 488, Goat, 1:500, Thermo Fisher Scientific, A-11039, AB_2534096
Chicken IgY-Alexa Fluor 488, Donkey, 1:500, Thermo Fisher Scientific, A78948, AB_2921070
Rat IgY-Alexa Fluor 568, Goat, 1:500, Thermo Fisher Scientific, A-11077, AB_141874
Rat IgY-Alexa Fluor 568, Donkey, 1:500, Thermo Fisher Scientific, A78946, AB_2910653
Guinea pig IgG-Alexa Fluor 488, Donkey, 1:500, Jackson ImmunoResearch, AB_2340472

Validation

GFP, Chicken, 1:500, Abcam, ab13970, AB_300798
Manufacturer's validation information: This antibody is suitable for WB, ICC/IF.
Selected citations: PMID: 34463618, PMID: 34292151

DCAMKL1, Rabbit, 1:250, Abcam, ab37994, AB_873538
Manufacturer's validation information: This antibody is suitable for ICC, WB, and IP.
Selected citations: PMID: 33649045, PMID: 35314700

cFOS, Rat, 1:300, Synaptic System, 226008, AB_2891278
Manufacturer's validation information: This antibody is suitable for WB, ICC, IHC, IHC-P, iDISCO, and Clarity.

Selected citations: PMID: 38368612, PMID: 38245542

CCK-8, Guinea pig, 1:500, Synaptic System,, 438004, AB_2814938
Manufacturer's validation information: This antibody is suitable for ICC, IHC, and IHC-P.
Selected citations: PMID: 39738072, PMID: 35550065

Eukaryotic cell lines

Policy information about cell lines and Sex and Gender in Research

Cell line source(s)	HEK293FT (Thermo Fisher Scientific, R70007)<br>This line is derived from the 293F Cell Line (originally obtained from Robert Horlick at Pharmacopeia) and stably expresses the SV40 large T antigen from the pCMVSPORT6TAg.neo plasmid.<br><br>R-spondin 1 expressing HEK293T (Sigma, SCC111)<br>This line is derived from the 293T cell line and stably expresses RSPO1, a protein used to establish 3D intestinal organoids.
Authentication	No authentication information could be found from the vendor's website
Mycoplasma contamination	No information about Mycoplasma contamination test could be found from the vendor's website
Commonly misidentified lines<br>(See ICLAC register)	N/A

Animals and other research organisms

Policy information about studies involving animals; ARRIVE guidelines recommended for reporting animal research, and Sex and Gender in Research

Laboratory animals	We used mice of both sexes between the age of 8-16 weeks. Mice were raised under regular diurnal (12:12) light-dark cycles at a temperature of 68-79 degrees F and a humidity of 30-70% with ad libitum access to food and water. Strains/genotypes used include:<br><br>Villin-Cre mice (MGI:2448639) from Jackson Laboratory<br>Tac1-IRES-Cre mice (MGI:5484668) from Jackson Laboratory<br>ePet1-Flp line (MGI:3795206) is a gift from Dr. Susan Dymecki.<br>RC::PFTox line (MGI:4412286) is a gift from Dr. Susan Dymecki.<br>Polr2aGCaMP5G-tdTomato mice (MGI: 5560331) from Jackson Laboratory<br>TRPM5-EGFP line is a gift from Dr. Robert Margolskee.<br>Ai32(RCL-ChR2(H134R)/EYFP) (MGI:5013789) from Jackson Laboratory.<br>Nav1.8-Cre was gifted from Dr. Wendy Imlach, Monash University, Australia. Jackson Laboratory, Strain no. 036564.<br>gGRAB5HT3.0-P2A-jRGECO1a mice were generated in Peking University (PMID: 39939779).<br>C57BL/6 mice bred at SAHMRI, aquired from Jackson Laboratory<br>Pou2f3-/- (Jackson Laboratory, Strain no. 037040).<br>Chatflox mice (gift from Dr. Jonah Chan).
Wild animals	The study did not involve wild animals.
Reporting on sex	We used mice of both sexes and pooled the data for analyses.
Field-collected samples	The study did not involve samples collected from the field.
Ethics oversight	All animal experiments done in UCSF were conducted in accordance with protocol AN192533 approved by the Institutional Animal Care and Use Committee, University of California – San Francisco. All animal experiments done in South Australian Health and Medical Research Institute (SAHMRI) were approved and performed in accordance with the guideline of the Animal Ethics Committees of SAHMRI.

Note that full information on the approval of the study protocol must also be provided in the manuscript.

Plants

Seed stocks	*Report on the source of all seed stocks or other plant material used. If applicable, state the seed stock centre and catalogue number. If plant specimens were collected from the field, describe the collection location, date and sampling procedures.*
Novel plant genotypes	*Describe the methods by which all novel plant genotypes were produced. This includes those generated by transgenic approaches, gene editing, chemical/radiation-based mutagenesis and hybridization. For transgenic lines, describe the transformation method, the number of independent lines analyzed and the generation upon which experiments were performed. For gene-edited lines, describe the editor used, the endogenous sequence targeted for editing, the targeting guide RNA sequence (if applicable) and how the editor was applied.*
Authentication	*Describe any authentication procedures for each seed stock used or novel genotype generated. Describe any experiments used to assess the effect of a mutation and, where applicable, how potential secondary effects (e.g. second site T-DNA insertions, mosiacism, off-target gene editing) were examined.*
